# Genome-Wide SNP Markers for Genotypic and Phenotypic Differentiation of Melon (*Cucumis melo* L.) Varieties Using Genotyping-by-Sequencing

**DOI:** 10.3390/ijms22136722

**Published:** 2021-06-23

**Authors:** Do Yoon Hyun, Raveendar Sebastin, Gi-An Lee, Kyung Jun Lee, Seong-Hoon Kim, Eunae Yoo, Sookyeong Lee, Man-Jung Kang, Seung Bum Lee, Ik Jang, Na-Young Ro, Gyu-Taek Cho

**Affiliations:** 1 National Agrobiodiversity Center, National Institute of Agricultural Sciences (NAS), Rural Development Administration (RDA), Jeonju 54874, Korea; raveendars@gmail.com (R.S.); gkntl1@korea.kr (G.-A.L.); lkj5214@korea.kr (K.J.L.); shkim0819@korea.kr (S.-H.K.); eung77@korea.kr (E.Y.); xsanta7@korea.kr (S.L.); mjkang@korea.kr (M.-J.K.); seungblee@korea.kr (S.B.L.); jangik5690@naver.com (I.J.); nonanona@korea.kr (N.-Y.R.); gtcho@korea.kr (G.-T.C.); 2 Honam National Institute of Biological Resources, Mokpo-si 58762, Korea

**Keywords:** genebank, GBS, genetic diversity, GWAS, muskmelon, varieties discrimination, SNP markers

## Abstract

Melon (*Cucumis melo* L.) is an economically important horticultural crop with abundant morphological and genetic variability. Complex genetic variations exist even among melon varieties and remain unclear to date. Therefore, unraveling the genetic variability among the three different melon varieties, muskmelon (*C. melo* subsp. *melo*), makuwa (*C. melo* L. var. *makuwa*), and cantaloupes (*C. melo* subsp. *melo* var. *cantalupensis*), could provide a basis for evolutionary research. In this study, we attempted a systematic approach with genotyping-by-sequencing (GBS)-derived single nucleotide polymorphisms (SNPs) to reveal the genetic structure and diversity, haplotype differences, and marker-based varieties differentiation. A total of 6406 GBS-derived SNPs were selected for the diversity analysis, in which the muskmelon varieties showed higher heterozygote SNPs. Linkage disequilibrium (LD) decay varied significantly among the three melon varieties, in which more rapid LD decay was observed in muskmelon (*r*^2^ = 0.25) varieties. The Bayesian phylogenetic tree provided the intraspecific relationships among the three melon varieties that formed, as expected, individual clusters exhibiting the greatest genetic distance based on the posterior probability. The haplotype analysis also supported the phylogeny result by generating three major networks for 48 haplotypes. Further investigation for varieties discrimination allowed us to detect a total of 52 SNP markers that discriminated muskmelon from makuwa varieties, of which two SNPs were converted into cleaved amplified polymorphic sequence markers for practical use. In addition to these markers, the genome-wide association study identified two SNPs located in the genes on chromosome 6, which were significantly associated with the phenotypic traits of melon seed. This study demonstrated that a systematic approach using GBS-derived SNPs could serve to efficiently classify and manage the melon varieties in the genebank.

## 1. Introduction

The melon, *Cucumis melo* L. (Cucurbitaceae), is an economically valuable horticultural fruit crop that is highly important in Mediterranean and East Asian countries. The world production of melons was estimated to be about 27.3 million tons from 1.04 million ha [1]. Based on the availability of many wild *Cucumis* specimens, Africa is believed to be the geographical origin of the melon [2]. However, based on recent taxonomical studies, both Africa and Asia have been proposed for species origins [3]. Similarly, the history of melon domestication and diversification is not yet clear [4]. African and Asian cultivars/landraces were clustered with wild types of their respective origins [5], since wild melons were found in both Africa and Asia. Archeological evidence suggests that melon domestication started ca. 5000–6000 years ago in Africa, which is earlier than Asia. Recently, [6] proposed two independent possible domestication events in Asia and Africa. However, there is still no considerable evidence of melon domestication processes; thus, it cannot be said where the melon was first domesticated.

In general, *C. melo* is considered to consist of two subspecies, *melo* and *agrestis* [7]. However, [8] divided the subspecies *melo* into ten groups and the subsp. *agrestis* into five groups, although these groups still display intermediate morphological features that are difficult to classify. Muskmelon is also known as *C. melo*, whereas the cantaloupe and makuwa are types of muskmelon, which causes concern with their differentiation. Oriental melon (*C. melo* L. var. *makuwa*), which is well-known as “chamoe” in Korea and was scientifically named after its origin village as the variety “makuwa”, is one of the most important annual diploid crops widely cultivated. It is primarily cultivated for its fruit in which fruit traits, such as fruit size, shape, skin color, and sugar content in the flesh, are highly variable [9]. Even though the morphological differences are easily distinguished between the muskmelon, cantaloupe, and makuwa at the fruit level, it is highly challenging to distinguish them at the plant or seed level. Hence, developing genotypic markers to differentiate muskmelon, cantaloupe, and makuwa at the DNA level among varieties will be helpful for differentiating varieties.

Molecular-based phylogenetic studies have been reported for the efficient classification of *C. melo* species, which clearly supports subspecies separation [3,5,10]. Based on seven DNA regions, [6] also reported a complex origin of the *C. melo* species from Africa, Asia, and Australia. In [11], the authors reported the phylogenetic classification of the species and subspecies. However, earlier studies have primarily focused on the phylogenetic relationships among species using inadequate taxon sampling or molecular markers with limited genomic information [12,13,14,15]. Single nucleotide polymorphism (SNP) markers were obtained with the aid of next-generation sequencing technologies to describe genetic diversity, as they appeared numerous throughout the genomic region with high reproducibility and were co-dominant in nature. The intraspecific classification of melon species was consistently improved with molecular marker-based phylogenetic studies, which supports the subspecies classification with sufficient differences [16].

The melon draft genome was recently released [17], which helped characterize genes related to melon domestication or selection, and many quantitative trait loci have been reported [18,19,20,21]. To understand the evolution of the melon genomic structure, a genome-wide association study (GWAS) was also performed [10,22,23,24]. However, difficulties in the intraspecific classification of the species occurred frequently due to the crossbreeding nature of the species. Moreover, the widespread occurrence of natural evolution from the wild type to landrace or improved varieties is not well understood, especially when breeders commercially improve cultivar/varieties.

Recently, genotyping-by-sequencing (GBS) approaches have been used as an efficient and affordable method for identifying a large number of genomic markers to differentiate species and subspecies [24,25]. In our earlier study, GBS-derived SNPs were efficiently used to classify *Triticum* species and subspecies that are very difficult to distinguish based on their morphological characteristics [26]. Genotyping-by-sequencing has also been used in the melon population, and highly informative genome-wide SNPs have been generated successfully [27,28]. To resolve the genetic variability between the muskmelon and makuwa varieties available in the Korean genebank, GBS-derived SNPs were mapped to the reference genome. The extent of linkage disequilibrium (LD) decay with distance in a population over time was influenced by the recombination rate between the SNP loci, which was used to understand the genome-wide variability in plants including melon [5,27,28]. Moreover, the GBS-generated SNPs were not only proven useful for studying germplasm diversity, population structure, and phylogenomics, but were also successful in GWAS.

According to the International Code of Nomenclature for Cultivated Plants (ICNCP), approximately 522 synonyms of *C. melo* have been recognized [29]. Therefore, there are many different types of melon available in the seed catalog that commonly come under the species name “*C. melo*”. Similarly, in the Korean genebank, all the melon accessions were commonly recorded as “*C. melo*”. Hence, it is impossible to differentiate seed accessions without standard passport descriptors. Moreover, all over the world, different melon varieties have been identified with the common name “melon”. However, in Korea, makuwa (*C. melo* L. var. *makuwa*) is generally called “chamoe” and treated as a different fruit from other melon species; people consume the fruit flesh and seeds of makuwa, which has smaller seeds than that of the melon. The seed size is an important characteristic to differentiate the makuwa from other melon varieties in terms of edibility. Hence, in the present study, we also attempted a GWAS to identify the SNPs associated with the phenotypic traits of melon seeds. The generated data provide new insights into the identification of candidate genomic regions that could be used to differentiate all three melon varieties in order to efficiently classify the melon accession resources in the genebank.

## 2. Results

### 2.1. GBS Analysis

To understand the genetic relationship between the three different melon varieties, 72 melon accessions consisting of muskmelon, makuwa, and cantaloupe were sequenced using GBS technology (Supplementary Materials Table S1). The sequencing data are presented in Supplementary Materials Table S2. Sequencing of the GBS library yielded 217.99 million (M) raw reads. After quality filtering, a total of 184.5 M clean reads with an average of 2.56 M reads per sample (ranging from 1.06 to 5.62 M) were generated from the raw reads. Statistical analysis of the sequence data further showed that the average quality value 30 (Q30) was ≥82.2%, indicating that the GBS library was sufficient for melon germplasm characterization. Each of the 72 sample reads was mapped to *Cucumis melo* L. cv. DHL92 v.3.5.1.

Among the GBS sequence reads, a total of 153.4 M reads with an average of 2.13 M (84.1%) reads were aligned to the reference genome. Among them, melon35 had the highest mapping rate (88%) and melon34 had the lowest rate (76.4%). Considering only the successfully mapped reads from the 72 melon accessions, SNPs were discovered by analyzing the single master alignment file and genotypes were named with GATK [30]. A total of 39,034 GBS-derived SNPs were identified and a total of 32,628 high-quality SNPs were filtered out from duplicated reads. Among them, 6406 SNPs with <5% missing data were selected. The homozygote and heterozygote SNP ratio across chromosomes showed that muskmelon (*C. melo* subsp. *melo*) has higher heterozygote SNPs (Supplementary Materials Figure S1). The number of homozygous SNP loci ranged from 1199 (melon18) to 3091 (melon59) and the heterozygote SNPs ranged from 90 (melon34) to 2835 (melon18) among the tested varieties (Supplementary Materials Table S3).

### 2.2. Genetic Structure and Molecular Diversity

An admixture-based clustering implemented in the STRUCTURE software and the DAPC were performed to infer the genetic structure of a germplasm collection. The STRUCTURE analysis results (Supplementary Materials Figure S2) revealed the best grouping number (K = 2) based on the delta K. Population 1 and 2 consisted of 44 and 17 accessions, respectively, and 11 accessions were identified in the admixed population (Supplementary Materials Figure S2).

**Figure 1:**
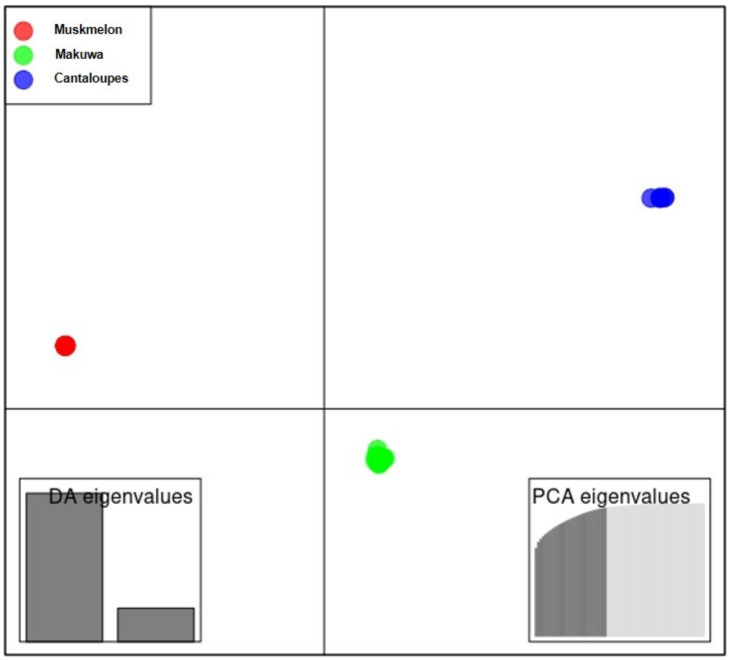
Discriminant analysis of principal components (DAPC) for 72 melon accessions using 6406 single nucleotide polymorphisms (SNPs). Eight first principal components (PCs) and three discriminant eigenvalues were retained during the analyses to describe the relationship between the clusters. The axes represent the first two linear discriminants (LD). Each circle represents a cluster and each color represent the different subpopulations identified by the DAPC.

Further, the DAPC was carried out to detect the possible number of clusters among the 72 accessions (Figure 1). The number of detected clusters was three, which coincided with the lowest BIC value obtained from the *find.clusters* function. Eight first PCs (53% of the variance conserved) of the PCA were retained, and three discriminant eigenvalues were confirmed by the cross-validation analysis. Clusters 1, 2, and 3 consisted of 18, 48, and six accessions, respectively. The distribution of the accessions in the three populations was fully matched with the classification of varieties as makuwa, muskmelon, and cantaloupe. Thus, each population was considered for genetic diversity analysis.

To quantify the genetic diversity of the three melon populations, Shannon’s diversity index (I) was employed using the GBS dataset. The I was 0.31 for pop1, 0.48 for pop2, and 0.47 for pop3 (Table 1). The number of effective alleles (Ne) was 1.31, 1.55, and 1.52, while the expected heterozygosity (He) was 0.19, 0.32, and 0.31 for pop1, pop2, and pop3, respectively. The percentage of polymorphic loci (%P) ranged between 83.7 and 96.8. The distribution of molecular variance among and within population clusters was estimated using AMOVA. The results reveal that based on pairwise PhiPT values, the genetic variability within clusters (54%) was greater than the variability among the clusters (46%) (Table 2). Pairwise PhiPT genetic distances (Table 3) ranged from 0.065 (Cluster 2/Cluster 3) to 0.549 (Cluster 1/Cluster 2), with a mean PhiPT value of 0.463, indicating significant variation among the population clusters (Table 2).

The genetic diversity among the 46 makuwa accessions was also assessed using diversity indices. The I was 0.11 for cultivar, and 0.10 for landrace varieties (Supplementary Materials Table S4). The number of alleles (Na) was 0.89 and 0.70, while the He was 0.07 and 0.06 for the cultivar and landrace varieties, respectively. The percentage of polymorphic loci (P%) was higher in the cultivar varieties (33.5%) than in landrace varieties (23.6%). The AMOVA results reveal that based on pairwise PhiPT values, the genetic variability within clusters (93%) was greater than the variability among the clusters (7%) (Supplementary Materials Table S5), with a mean PhiPT value of 0.074, indicating considerable variation between clusters (Supplementary Materials Table S5).

### 2.3. LD Decay

**Figure 2:**
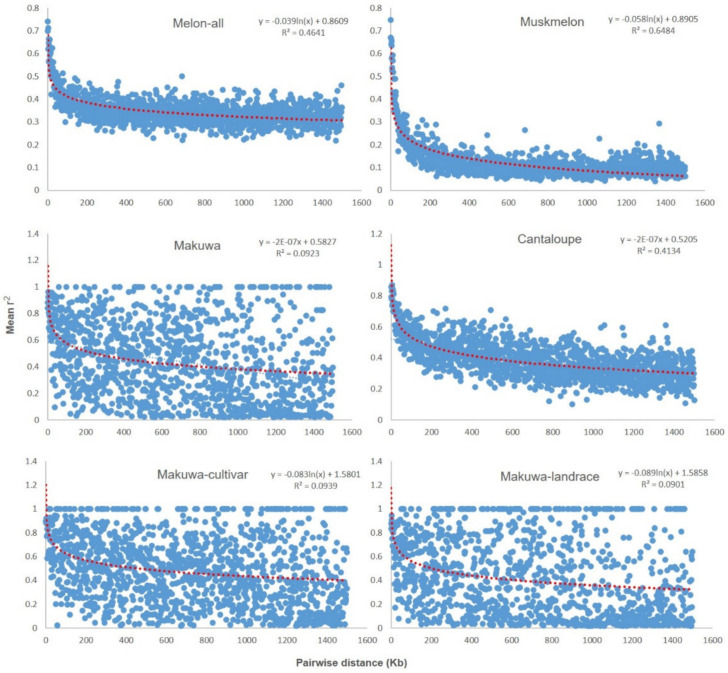
Linkage disequilibrium (*r*^2^) versus physical distance (kb) in the whole melon accessions collection. Genome-wide pattern of decay of linkage disequilibrium (LD) up to 1500 kb pairwise distances.

This study showed three distinct populations in the *C. melo* germplasm collection; we decided to estimate the LD decays separately (Figure 2). The LD was highly variable among the different genomic windows. The LD decay was clearer with the pairwise distance, in which the threshold value reached *r*^2^ < 0.4 at 100 kb when the LD was analyzed among all varieties. The LD was also calculated separately for the makuwa, muskmelon, and cantaloupe varieties defined by the DAPC. As the LD varied significantly among the three varieties (Figure 2 and Table 4), the LD decay distance (to *r*^2^ = 0.5) for makuwa and cantaloupe was approximately 200 and 100 kb, respectively. For muskmelon, the LD decay distance was approximately 50 kb (*r*^2^ = 0.25). As the threshold value of the LD decay was very high for the makuwa (*r*^2^ < 0.5), we decided to estimate the LD decays between cultivar and landraces. The results reveal that the landraces decayed faster than the cultivar varieties (Figure 2 and Table 4).

### 2.4. Phylogeny for Discrimination of Varieties

**Figure 3:**
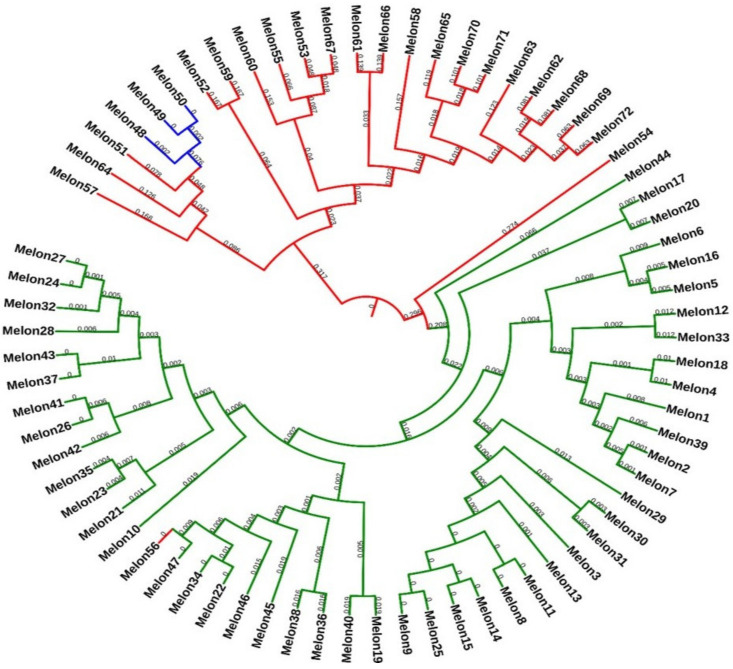
Bayesian phylogenetic tree of 72 accessions of the melon varieties using 6406 single nucleotide polymorphisms (SNPs) (<5% missing data) obtained by genotyping-by-sequencing (GBS). Each color represents three different melon varieties. Numbers in nodes are Bayesian posterior probabilities.

A Bayesian phylogenetic tree for all 72 accessions was constructed for a better visualization of their relationships. The Bayesian phylogenetic reconstruction of melon varieties showed a highly resolved phylogeny (Figure 3). In the Bayesian tree, all three melon varieties (makuwa, muskmelon, and cantaloupe) formed individual clusters where a single melon (subsp. *melo*) accession (melon56) was clustered together with the makuwa clade, and a dudaim melon accession (melon54) was located closer to the makuwa clade; this was similar to the results of the ADMIXTURE (Figure 3). The phylogenetic tree clearly provided the intraspecific relationships between the three melon varieties. As expected, based on the posterior probabilities, the three examined varieties were clustered separately from each other, while muskmelon and cantaloupe varieties were found in the same clade.

### 2.5. Haplotype Network

**Figure 4:**
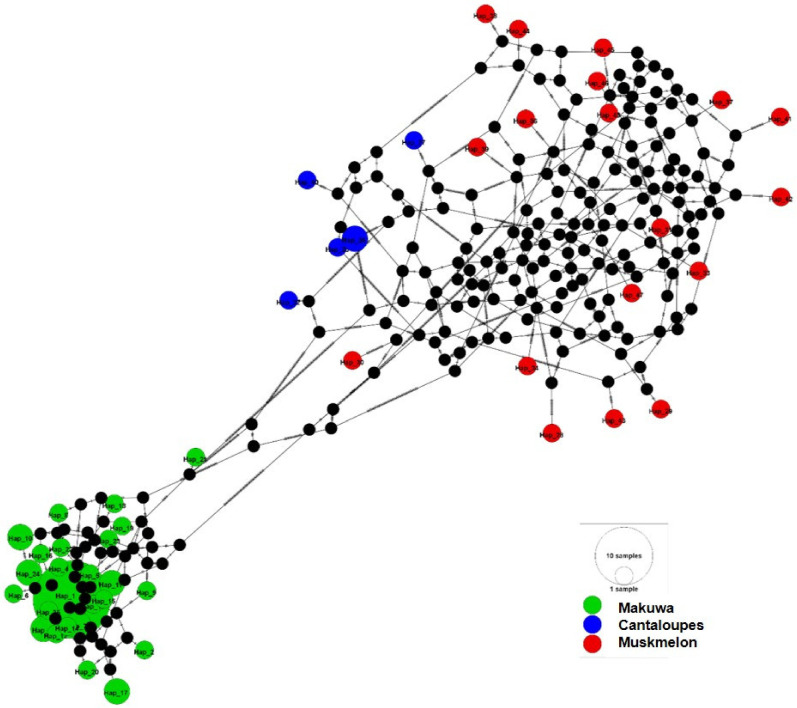
Haplotype network analysis calculated for 72 melon accessions using the integer neighbor-joining haplotype network. The integer neighbor-joining network was generated in PopArt with the reticulation tolerance set to the default value of 0.5. Individual hatch marks on the lines connecting haplotypes indicate mutations. The black dots indicate inferred intermediate haplotypes.

The concatenated SNP matrix exhibited a total of 48 haplotypes among the varieties. Using an integer neighbor-joining network [31], we attempted to draw the three observed haplotypes from those of the extant neighboring populations. The integer neighbor-joining haplotype network revealed three major networks (Figure 4), with a clear distinction among makuwa, muskmelon, and cantaloupe haplotypes.

### 2.6. Evaluation of SNP Markers for Varieties Discrimination

The difference between muskmelon and makuwa varieties were found to be difficult to distinguish because of their genotypic relationship. Initially, a total of 6406 SNPs were filtered from the raw variants to discriminate the muskmelon, makuwa, and cantaloupe varieties. Furthermore, in a Pearson’s chi-squared test, a total of 52 SNPs specific to each variety were detected based on allele frequencies. The concatenated consensus SNP markers showed clear discrimination of makuwa, muskmelon, and cantaloupe varieties (Supplementary Materials Figure S3 and Table S6), where the var. *cantalupensis* was also located closer to muskmelon accessions as a result of phylogeny. Similarly, a dudaim melon accession (melon54) and a misidentified accession (melon56) in the Bayesian phylogeny also showed a clear variation between makuwa and muskmelon accessions.

### 2.7. Development of CAPS Markers

**Figure 5:**
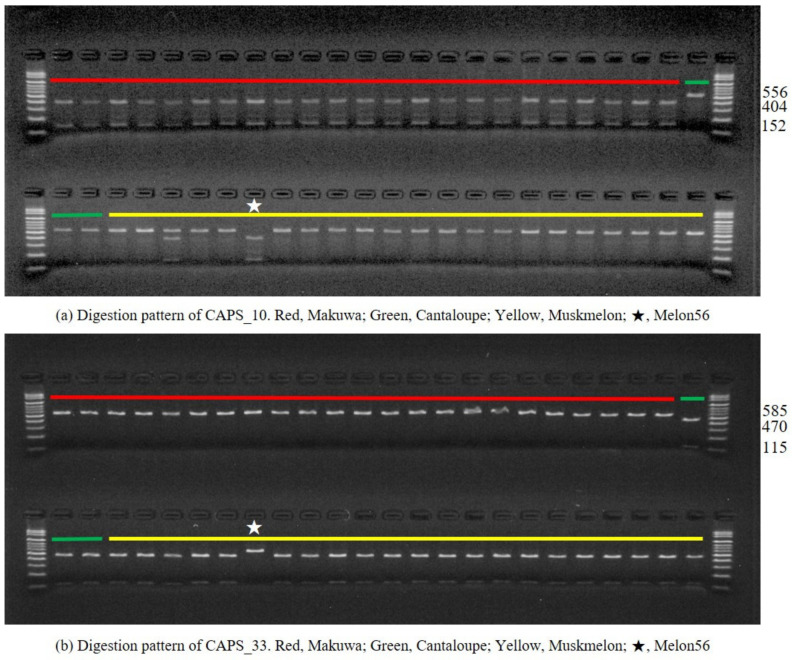
Gel electrophoretic image of cleaved amplified polymorphic sequence (CAPS) polymorphism of polymerase chain reaction products digested with (**a**) *Bcc*I and (**b**) *Bsr*I.

To discriminate the melon varieties for the efficient management of melon accessions in the genebank, the SNPs were converted into CAPS markers. The developed CAPS markers were tested on 23 representative makuwa accessions along with muskmelon and cantaloupe accessions (Figure 5). Among the 52 SNPs selected, two SNP positions identified in the intergenic region, namely 27,668,340 bp in chromosome 3 (MELO3C010934) and 22,254,315 bp in chromosome 9 (MELO3C005675), were successfully recognized with the restriction enzyme. The SNP located on chromosome 3 had a recognition site (GGTAG) for the *Bcc*I restriction enzyme, which was developed as CAPS_10. The PCR product (556 bp) was digested with the *Bcc*I enzyme, which produced an uncut allele (556 bp) pattern in muskmelon and cantaloupe, whereas a digested allele (152 and 404 bp) pattern was produced in makuwa (Figure 5a). Moreover, the muskmelon accession (melon56) had a similar digested allele pattern to makuwa. Similarly, the SNP located on chromosome 9 had a recognition site (TGACC) for the *Bsr*I restriction enzyme, which was developed as CAPS_33. The PCR product (585 bp) was digested with *Bsr*I, which produced the opposite allele pattern of CAPS_10. Muskmelon and cantaloupe varieties produced a digested allele (115 and 470 bp), whereas makuwa produced an uncut allele (585 bp). Likewise, the muskmelon accession melon56 produced an uncut allele pattern similar to makuwa (Figure 5b).

### 2.8. Identification of Genes or Loci Related to Agronomic Traits

**Figure 6:**
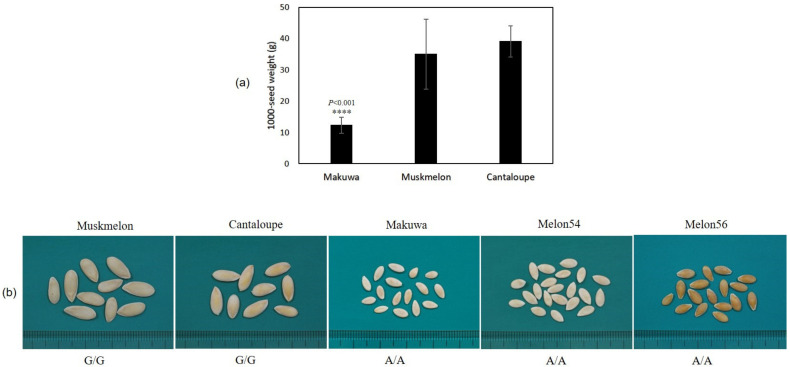
Variation in agronomic trait of 1000-seed weight (TSW) among melon varieties (**a**) and the genotype frequency at the single nucleotide polymorphism (SNP) locus S6_5912593 (**b**) in the melon varieties of the present study.

To identify the causative genes for the agronomic traits, we performed an association study with mixed models using a panel composed of 72 accessions for the agronomic traits of seed phenotypic descriptors (Supplementary Materials Table S1). When we compared the phenotypic characteristics of the seeds (such as TSW, length, and width) among varieties, makuwa was smaller than muskmelon and cantaloupe accessions (Figure 6). However, based on the phenotypic characteristics, dudaim melon (melon54) and muskmelon (melon56) accessions were smaller in size when compared with other muskmelon accessions (Supplementary Materials Table S1), which creates more deviation in TSW within muskmelon accessions (Figure 6).

Manhattan plots of the association analysis revealed strong signals for the phenotypic traits of melon seeds (Supplementary Materials Figure S4). Among the signals, a total of four SNPs (S6_875904, *p* = 0.00016; S6_5912593, *p* = 0.00042; S8_11953060, *p* = 0.00002; S9_23627273, *p* = 0.00009) on chromosomes 6, 8, and 9 were significantly associated with the TSW of melon seeds. However, SNPs located on chromosomes 8 and 9 were predicted to be a hypothetical protein and S-type anion channel SLAH2, splicing intron, respectively, whereas the SNPs S6_875904 and S6_5912593 were located in the genes annotated as protein ABIL 1 and titin homolog isoform x2, respectively. A set of 11 and 12 associated signals which spanned 275.25 (from 615,394 to 890,641 bp) and 369.07 kb (from 5,600,665 to 5,969,733 bp), respectively, were also identified around these two SNP regions in the melon DHL92 V3.5.1 reference genome (Supplementary Materials Figure S5). Various genes predicted for the seed ontology were detected in these regions. The LD haplotype analysis with the 72 accessions showed associations of S6_875904 and S6_5912593 with 11 and 12 other loci, respectively, which revealed LD blocks in these regions (Supplementary Materials Figure S5a,b).

Manhattan plots of the association analysis revealed strong signals for the phenotypic traits of melon seeds (Supplementary Materials Figure S4). Among the signals, a total of four SNPs (S6_875904, *p* = 0.00016; S6_5912593, *p* = 0.00042; S8_11953060, *p* = 0.00002; S9_23627273, *p* = 0.00009) on chromosomes 6, 8, and 9 were significantly associated with the TSW of melon seeds. However, SNPs located on chromosomes 8 and 9 were predicted to be a hypothetical protein and S-type anion channel SLAH2, splicing intron, respectively, whereas the SNPs S6_875904 and S6_5912593 were located in the genes annotated as protein ABIL 1 and titin homolog isoform x2, respectively. A set of 11 and 12 associated signals which spanned 275.25 (from 615,394 to 890,641 bp) and 369.07 kb (from 5,600,665 to 5,969,733 bp), respectively, were also identified around these two SNP regions in the melon DHL92 V3.5.1 reference genome (Supplementary Materials Figure S5). Various genes predicted for the seed ontology were detected in these regions. The LD haplotype analysis with the 72 accessions showed associations of S6_875904 and S6_5912593 with 11 and 12 other loci, respectively, which revealed LD blocks in these regions (Supplementary Materials Figure S5a,b).

## 3. Discussion

### 3.1. Evaluation of SNP Characteristics

Various molecular markers have been used extensively in genetic diversity analyses to characterize the plant germplasm over the past two decades [32]. Recently, GBS technology has become a powerful method for studying the genetic characteristics of plant species [33]. Similarly, the GBS based genotyping strategies have also been used for the analysis of melon genotypic variability [11,23]. In the present study, we analyzed the genetic variability of 72 melon accessions using 32,628 GBS-derived SNPs. Compared with the GBS-derived SNP results in previous studies [27,28], this study collected three different varieties that yielded more SNPs. Finally, 6406 SNPs with < 5% missing data were selected for further study. Recently, Moing et al. [34] reported the infraspecific classification of *C. melo* cultivar groups based on a combination of about > 80,000 metabolomic features together with >20,000 SNPs. Likewise, GBS combined with SNP validation assays has also been tested in commercial melon cultivars identification using 9018 GBS-derived SNPs [35]. Similarly, in the present study, 32,628 GBS-derived SNPs allowed us to infer infraspecific classification among the *C. melo* varieties of the widely cultivated species. The heterozygous SNP ratio across chromosomes showed that the muskmelons (*C. melo* subsp. *melo*) have a greater range of heterozygous markers than the makuwa (*C. melo* L. var. *makuwa*) variety (Supplementary Materials Figure S1). More heterozygous markers in the muskmelon variety could be due to their outcrossing nature, as reported previously [36].

### 3.2. Population Structure and Genomic Variability

The model-based STRUCTURE analysis classified 72 melon accessions into two groups (Supplementary Materials Figure S2). In addition, 72 melon accessions were divided into three well-defined clusters, which were clearer than their genetic structure in the result of the DAPC (Figure 1). The fact that muskmelon, makuwa, and cantaloupe accessions were grouped distinctly indicated that these accessions had the highest level of genetic variability. Our study clearly differentiated muskmelon, makuwa, and cantaloupes into different clusters, which is in agreement with previous studies [12,13,16,37,38,39,40,41]. Recently, Nimmakayala et al. [22] analyzed 120 melon accessions, which contained a good representative collection of melon species and showed clear differentiation of melon species in which makuwa and cantaloupe varieties were clustered separately.

To resolve the differentiation among muskmelon, makuwa, and cantaloupe, we estimated pairwise (PhiPT) values across all polymorphisms with MAF ≥ 0.05 (Table 1). All PhiPTs were highly significant (*p* < 0.001). The PhiPT value between accessions of muskmelon and makuwa was 0.549, whereas that between makuwa and cantaloupe was 0.301. Cantaloupe was found to be much closer to muskmelon than the makuwa varieties, with a PhiPT value of 0.065. The AMOVA revealed that based on pairwise PhiPT values, the genetic variability within clusters (54%) was greater than the variability among the clusters (46%) (Table 2). Pairwise PhiPT genetic distances (Table 3) between populations indicated significantly high variation among population clusters (Table 2). Similarly, the He varied from 0.191 to 0.318, which suggests the extent of variation between the three varieties, as reported in previous studies [22,42].

### 3.3. LD Decay and Haplotype Network

To conclude the genome-wide LD, a high-density SNP array should be analyzed [43]. The current results reveal that the LD was high, in the range of kb, when analyzing samples from all melon accessions (Figure 2 and Table 4). For an in-depth analysis of genome-wide experiments, one SNP per every kb or lower density would be necessary to ensure the detection of LD decay. Therefore, the extent of LD in melon is similar to that reported in other species, such as tomato [44,45], wheat [46], peach [47], barley [48], and rice [49]. On the other hand, the current results show that muskmelon alone decays rapidly, within 50 kb. Previous studies on the LD decay for melon populations showed that the LD decays more rapidly within a few kilobases, which might be due to the use of very different germplasms [5,27,28].

In general, the LD declines more slowly in self-pollinated crops, where recombination is less effective than in cross-pollinating species [50,51]. Higher LD levels were also found in flax [52] and sesame [53] because of self-pollination. We found the slowest LD decay was in makuwa, as the level of genetic variation found within the varieties influenced the extent of LD, where LD decay was rapid in landrace accessions compared to related cultivars, as reported in other species [54]. The much lower *r*^2^ value and longer LD distance for the makuwa and cantaloupe suggested that these varieties may have undergone a severe bottleneck.

To describe the genetic structure of the melon populations, we performed haplotypes using integer neighbor-joining network analysis (iNJ). The iNJ network revealed three major networks (Figure 4), with a clear distinction between muskmelon, makuwa, and cantaloupe haplotypes. The concatenated SNP matrix exhibited a total of 48 haplotypes, in which makuwa showed higher haplotype frequencies (52%), followed by muskmelon (37.7%) and cantaloupe (10.4%) varieties. Esteras et al. [6] also recognized three clades based on the median-joining network with an ITS dataset containing wild and cultivar melon accessions from Africa, Asia, the Mediterranean, and Australia.

### 3.4. Phylogeny for Discrimination of Melon Varieties

In general, the evolutionary relationship between species is revealed through phylogenetic analysis. In an earlier study, Pitrat [8] divided the subspecies *melo* into ten groups, and the subsp. *agrestis* into five groups. However, some of these accessions displayed intermediate features and were difficult to classify. Early taxonomic work failed to separate the cultivated species from wild species, resulting in approximately 522 synonyms *of C. melo* species in the seed catalog [29]. Therefore, around the globe, wild as well as cultivated melon varieties have been recorded as “*C. melo*” and commonly identified as “melon”. Similarly, in the Korean genebank, all the melon accessions were recorded as “*C. melo*”, which makes them difficult to differentiate without standard passport data. Therefore, the present study aimed to test the accuracy of varieties discrimination for a total of 72 accessions, including makuwa (*C. melo* L. var. *makuwa),* muskmelon (*C. melo* subsp. *melo*)*,* and cantaloupe (*C. melo* subsp. *melo* var. *cantalupensis*) with 6406 genome-wide SNP markers. The Bayesian phylogeny tree clearly showed that makuwa and muskmelon accessions were clustered in an individual clade (Figure 3). The Bayesian tree showed cantaloupe accessions clustered together with the muskmelon (*C. melo* subsp. *melo*) population, which was similar to the STRUCTURE and iNJ networks. In a recent study, a total of 23,931 GBS derived SNPs successfully classified 44 melon accessions into two well-defined clusters, which clearly distinguished between the subspecies *agrestis* and *melo* [34]. However, a charentais-type *Cantalupensis* melon accession was placed closer to the subspecies *melo*, and a dudaim melon accession was placed between the subspecies, which coincides with the present study, where the variety of cantaloupes and a dudaim melon accession clustered, respectively. It is believed that cantaloupe melons originated from South Asia to Africa and spread to Europe [55]. Moreover, the cantaloupes, comprising many cultivated varieties from Europe, Asia and America [56], are more diverse than makuwa. However, based on the present study, a muskmelon accession (melon56) was misidentified or incorrectly classified, which requires critical evaluation.

### 3.5. SNP Markers for Varieties Differentiation

Single nucleotide polymorphisms are valuable markers for discovering species relationships; however, it is highly challenging to validate a subset of melon accessions [57]. Various high-throughput genotyping assay techniques have been developed and successfully used in land plants [58,59,60]. However, these high-throughput methods may not be suitable for germplasm management in genebanks where a large number of accessions are conserved. To minimize the effort of high-throughput genotyping assays, developing SNP markers could be a better solution.

Analysis revealed a total of 52 SNPs based on allele frequencies. The concatenated consensus SNPs clearly discriminated between makuwa and muskmelon accessions (Supplementary Materials Figure S3 and Table S6). Similarly, the cantaloupe accessions also showed 96% variation with makuwa, whereas there was only 4% variation with muskmelon varieties, which was similar to STRUCTURE and Bayesian phylogeny. Moreover, the two melon accessions (melon54 and melon56) also revealed the reason behind their clustering with the varieties of makuwa. Among the 52 SNPs, the melon54 accession showed 51 non-matching SNPs with makuwa (98%), whereas there were only six non-matching SNPs with muskmelon varieties (11.5%). Similarly, melon56 accessions showed five non-matching SNPs with makuwa (9.6%), whereas there were 49 non-matching SNPs with muskmelon varieties (94.2%), which was repeated in the Bayesian phylogeny. Hence, the present study suggests that the melon54 accession (dudaim melon) is more appropriate for muskmelon (*C. melo* subsp. *melo*), whereas the melon56 accession belongs to the variety makuwa (*C. melo* L. var. *makuwa*), which requires more critical evaluation at the field level.

### 3.6. Validation of CAPS Markers

Various SNP-based molecular markers have been developed and successfully used in plant species identification [5,26,53,60]. Among these, CAPS markers have been found to be promising for detecting the intra- and interspecies variation of different species [61]. Similarly, in the present study, two intergenic SNP positions, CAPS_10 and CAPS_33, were found to be promising loci for discriminating the melon varieties. In mammalian systems, most intergenic transcripts were found to be un-spliced and associated with nearby gene expression [62]. Moreover, the intergenic transcribed regions found to be more divergent in expression tended to be more species-specific when compared to annotated genes across plant species [63]. When the PCR products (556 and 585 bp) were digested with the respective enzymes (*Bcc*I and *Bsr*I), they produced distinct allele patterns between the muskmelon and makuwa populations (Figure 5). Interestingly, the muskmelon accession melon56, which was clustered together with the makuwa accessions in the phylogenetic tree, showed a very similar allele pattern to that of makuwa, which requires critical evaluation within the GMS in order to manage the melon accessions correctly in the genebank.

### 3.7. Identification of Agronomic Traits

Certainly, all over the world, seedless characteristics always improve the economic value of fruits. In Korea, people prepare makuwa (chamoe) to consume the fruit flesh, including the seeds, as the seeds are tiny compared with melon seeds. Hence, seed size is an important characteristic for differentiating the makuwa variety from muskmelon in terms of edibility. Various studies on genes or loci underlying agronomic traits have been reported in melon [10,22,23]. In a previous study, Pavan et al. [28] detected significant associations between seed width and flowering time. Interestingly, a candidate gene (*MSI1*) associated with seed development [64] was detected for seed width. Similarly, in the present study, to identify the causal genes for an agronomic trait, we performed an association study with the phenotypic traits of melon seeds. Functional analysis of candidate genes identified in this study could be useful to confirm the link of phenotypic variation in melon.

Based on phenotypic traits, makuwa has a smaller seed weight than the muskmelon and cantaloupe accessions (Figure 6). However, interestingly, the TSW of dudaim melon (melon54) and muskmelon (melon56) accessions showed smaller seed weights (13 and 8.4 g), which were similar to makuwa TSW (Supplementary Material Table S1). The GWAS results reveal phenotypically associated signals within the melon genome (Supplementary Materials Figure S4), in which two SNPs on chromosome 6 (S6_875904 and S6_5912593) were significantly associated with the phenotypic traits of melon seeds (Supplementary Materials Figure S5a,b). Both SNPs were located in the genes predicted for seed ontology, in which the annotated protein ABIL1 and titin homolog isoform x2 were reported for seed development [65,66].

The SNP (S6_875904) located on the protein ABIL1 coding region was found to be missense, which makes the stop codon of the transcript, whereas the SNP (S6_5912593) located on the titin homolog isoform x2 was found to be synonymous. Genome-wide analysis of the maize genome reveals that synonymous mutations change tRNA adaptation, which affects the local translation rate [67]. There is much experimental evidence of the synonymous mutation effects on the phenotypes of different organisms [68,69,70]. Moreover, the SNP S6_5912593, identified to be an A/A haplotype similar to makuwa within the dudaim melon (melon54) and muskmelon (melon56) accessions, revealed that the gene titin homolog isoform x2 could play a crucial role in seed development, as reported earlier in Arabidopsis. These markers, combined with SNP markers, can be used to manage the melon accessions in order to provide accurate information in the genebank.

## 4. Materials and Methods

### 4.1. Plant Materials

For this study, a total of 72 melon accessions were obtained from the Korean genebank of the National Agrobiodiversity Center at the Rural Development Administration in South Korea (Supplementary Materials Table S1). Based on seed metadata information, 21 muskmelon and a dudaim melon, also called “wild muskmelon”, accessions were introduced from USDA-ARS and were collected from different countries. Since all the melon accessions were commonly recorded as “*C. melo*”, hereafter we would like to classify these melon accessions into the following three varieties: muskmelon (*C. melo* subsp. *melo*), makuwa (*C. melo* L. var. *makuwa*)*,* and cantaloupe (*C. melo* subsp. *melo* var. *cantalupensis*), which could be further classified into cultivar and landraces according to the germplasm introduction information. Seed phenotypic data such as 1000-seed weight (TSW), length (cm), and width (cm) were retrieved from the genebank management system (GMS) of the genebank for the association mapping analysis with each accession.

### 4.2. DNA Extraction

For the GBS of the melon collection, 30 mg of freeze-dried leaf tissue was taken from the 72 accessions listed in Supplementary Table S1. Total genomic DNA isolation was performed according to the manufacturer’s recommendation using the QIAGEN plant mini kit (Qiagen, Valencia, CA, USA). The quality of DNA in each sample was determined using 1% (*w*/*v*) agarose gel electrophoresis and quantified by spectrophotometry.

### 4.3. Preparation of Genotyping-by-Sequencing Libraries

The extracted DNA was quantified and normalized to 12.5 ng/µL using Quant-iT PicoGreen dsDNA Assay Kit (Molecular Probes, Eugene, OR, USA) with a Synergy HTX Multi-Mode Reader (Biotek, Winooski, VT, USA). The DNA was treated with the restriction enzyme *Ape*KI (New England Biolabs, Ipswich, MA, USA) at 75 °C for 3 h. Sequencing libraries for GBS were constructed according to previously described procedures [25]. The DNA samples were digested and ligated with adapters, which contained different barcodes for tagging individual samples. Ligated samples were pooled and purified with a NucleoSpin^®^ Gel and polymerase chain reaction (PCR) Clean-up Kit (MACHEREY-NAGEL GmbH & Co. KG, Duren, Germany). The purified samples were PCR amplified in a 50 µL reaction and the amplified products were evaluated for fragment sizes using BioAnalyzer 2100 (Agilent Technologies, Santa Clara, CA, USA). Illumina NextSeq500 (Illumina, San Diego, CA, USA) was used to sequence the GBS libraries with a read length of 150 bp single-end reads.

### 4.4. Sequence Preprocessing and SNP Calling

The sequence reads were preprocessed initially in three stages: demultiplexing, per-base quality control, and removal of adaptor using Stacks, FastQC, and Cutadapt software [71,72,73]. High-quality reads were then mapped to the *Cucumis melo* L. cv. DHL92 v.3.5.1. reference genome using Bowtie2 [74]. The GenomeAnalysis Toolkit, GATK, V 3.3-0 [30], was used to call high-quality SNPs with the following criteria: duplicate reads were removed using Picard package V1.105 and the base quality score was recalibrated by the Base Recalibrator package in GATK. Finally, the SNPs were called using the UnifiedGenotyper function in GATK with the following parameters: quality score (QUAL < 30), quality depth (QD < 5), Fisher score (FS > 200), and with vcftools v. 0.1.15 to restrict the maximum missing rate (--max-missing 0.95), allele frequency (--maf 0.05), allele number (--min-alleles 2, --max-alleles 2), and read depth for the SNP locus (--min-meanDP 5).

### 4.5. Population Structure and Genetic Diversity

The discriminant analysis of principal components (DAPC) was used to assign the individual accessions to the population clusters [75]. The DAPC requires the construction of prior groups; therefore, the most likely number of clusters in each melon was identified by the “*find.clusters*” function in the R package *adegenet*, based on the Bayesian Information Criterion (BIC). In the DAPC analysis, a two-step procedure was followed in which the original data were transformed and submitted to a principal component analysis (PCA), and the principal components (PCs) were passed to a linear discriminant function analysis based on the groups identified in the K-means clustering. The PCs retained well with the population numbers, leading to accurate discriminant functions, resulting in perfect discrimination [76]. Hence, “*optim.a.score*” function was used to assesse the quality of discrimination that served as the best criteria to choose the optimal number of PCs in the DAPC [75]. The resulting clusters were plotted as a DAPC scatterplot with the first and second linear discriminants.

To investigate the population structure, admixture analysis was performed on the 72 individuals using the ADMIXTURE tool (available from: http://software.genetics.ucla.edu/admixture/index.html, accessed on 12 April 2021). The admixture-linux-1.3.0 was run with default parameters in an unsupervised mode of *K* = 1 to 21. The cross-validation error for each *K* was computed with the -cv option (10 folds), which identified *K* = 2 as the most suitable modeling choice.

For each melon population, we used hierarchical analyses of molecular variance (AMOVA) to investigate the molecular variation within and among the groups defined by the DAPC function. The AMOVA and the pairwise genetic differentiation (PhiPT) between and among melon varieties were calculated using GenAlEx software (6.5 version) with 999 permutations [77]. Expected heterozygosity (He), unbiased expected heterozygosity (uHe), and the percentage of polymorphic loci were also calculated using GenAlEx software.

### 4.6. Linkage Disequilibrium Decay and Haplotype Analysis

To understand the genome-wide variability among the three varieties, pairwise estimates of linkage disequilibrium (LD) were measured by the squared correlation analysis of allele frequencies (r^2^) with minor allele frequency (MAF) > 0.05. In addition, an LD threshold (r^2^) of 0.20 with a window size of 100 kb was used to calculate the correlation coefficient (r^2^) of alleles using the software PopLDdecay [78]. The LD was analyzed for different sub-datasets: total population, makuwa, muskmelon, and cantaloupe-related groups as defined by DAPC. The LD decay with distance in base pairs (bp) between sites within the candidate locus was evaluated using a regression curve. The haplotype frequency within the population groups was calculated using Arlequin software Ver. 3.5.2.2. The phylogenetic network was performed using an integer neighbor-joining network [31] with popART (http://popart.otago.ac.nz, accessed on 7 April 2020).

### 4.7. Phylogenetic Relationships

All SNPs were concatenated into a single alignment. Beast v2.1 was used to calculate the score for the substitution of SNPs, and Bayesian analyses were conducted with the GTR + G nucleotide substitution model using MrBayes version 3.2.6. The GTR + G model was chosen in both the AIC and hLRTs models for the model estimation. The model was estimated by MrModelTest version 2.4, using the calculated score as the input value [79]. In the Bayesian analyses, trees were sampled every 1000 generations using MrBayes until the average deviation of the split frequencies fell below 0.01 [80].

### 4.8. Evaluation of SNP Markers for Varieties Discrimination

Initially, to identify the SNP markers, fine SNPs were filtered from the raw variants to discriminate the muskmelon, makuwa, and cantaloupe varieties. Further specific SNPs were filtered based on the allele frequency between varieties to discriminate them. Pearson’s chi-squared test was performed to identify the significant SNPs that discriminated melon varieties.

### 4.9. CAPS Marker Development

To validate the SNPs for variety discrimination, cleaved amplified polymorphic sequence (CAPS) markers were developed with the information of 52 SNPs. The web-based program dCAPS finder 2.0 (www.helix.wustl.edu/dcaps, accessed on 11 June 2020) was used to find the restriction enzyme sites within the SNP position. To detect SNPs retained in the melon varieties, amplification reactions were carried out using appropriate primers. The PCR product obtained from the amplification of specific SNP regions was digested with 1U of restriction enzyme (New England BioLabs, Ipswich, MA, USA). Digestion was performed at 37 °C (*Bcc*I) or 65 °C (*Bsr*I) for 1 h, and the fragments were analyzed with 2.0% agarose gel electrophoresis.

### 4.10. Genome-Wide Association Mapping

Genome-wide association analysis for phenotypic traits of seeds was performed with 6406 high-quality SNPs (MAF > 0.05). Here, GAPIT implemented a series of methods for GWAS and genomic selection for high statistical power, high prediction accuracy, and high computing speed [81]. To perform the GWAS with maximum accuracy, mixed models were chosen, which included the general linear model (GLM), mixed linear model (MLM), multilevel mixed model (MLMM), fixed and random model circulating probability unification (FarmCPU), and Bayesian-information and linkage-disequilibrium iteratively nested keyway (BLINK). The *p*-values of the correlation association of each SNP with agronomic traits were calculated with the GAPIT R package [82]. The LD heatmap and regional association statistics for TSW were analyzed with LDBlockShow [83] together with the publicly available *Cucumis melo* L. cv. DHL92 v.3.5.1. genome browser (http://cucurbitgenomics.org/, accessed on 15 June 2021).

## 5. Conclusions

Highly informative SNP markers were developed in the present study through GBS analysis. The identified SNP markers provide a clear picture of the genomic relationships among the collection of the 72 melon accessions using a set of 6406 genome-wide SNPs. The DAPC and population structure seem to be defined mainly by their varieties. The cantaloupe (*C. melo* subsp. *melo* var. *cantalupensis*) varieties were closer to muskmelon (*C. melo* subsp. *melo*) than makuwa (*C. melo* L. var. *makuwa)*. Bayesian phylogeny of the melon varieties showed a highly resolved phylogeny, and the developed SNP markers clearly discriminated the corresponding varieties more accurately. The SNP markers could be standardized easily with a very low cost and minimum equipment for quick operation in a genebank. In association mapping, two SNPs on chromosome 6 were significantly associated with the phenotypic traits of melon seeds. The SNP variations of protein ABIL1 and titin homolog isoform x2 could be used in molecular breeding to develop commercially improved cultivars/varieties. Overall, this study provides a systematic approach for the efficient classification of melon seed accessions using genome-wide information. Information on genomic variability between melon varieties will facilitate the efficient classification and utilization of these resources in the genebank.

## Figures and Tables

**Table 1:** Statistics of the genetic variation for the melon populations.

POP	N	Na	Ne	I	He	uHe	%P
1	48	1.771	1.310	0.306	0.191	0.193	87.28%
2	18	1.937	1.540	0.481	0.318	0.318	96.52%
3	6	1.726	1.517	0.463	0.310	0.310	85.14%

N, number of individuals; Na, number of alleles; Ne, number of effective alleles; I, information index; He, expected heterozygosity; uHe, unbiased expected heterozygosity; %P, percentage of polymorphic loci.

**Table 2:** Results of the analysis of molecular variance (AMOVA) within and among the groups of 72 melon accessions identified by the discriminant analysis of principal components (DAPC) clustering.

SV	df	SS	MS	Est. Var.	%	PhiPT
Among Pops	2	24,193.625	12,096.813	648.326	46%	0.463
Within Pops	69	51,826.444	751.108	751.108	54%	
Total	71	76,020.069		1399.434	100%	

SV, source of variation; df, degrees of freedom; SS, sum of squares; MS, mean square; Est. Var., estimated variance; %, percentage of variation.

**Table 3:** Pairwise genetic differentiation values among the clusters.

Cluster	Cluster	PhiPT
1	2	0.549
1	3	0.301
2	3	0.065

**Table 4:** Mean linkage disequilibrium (LD) values according to distance (kb). The LD was calculated separately for the makuwa, muskmelon, and cantaloupe groups defined by the discriminant analysis of principal components (DAPC).

Distance (kb)	Overall LD		Makuwa		Muskmelon		Cantaloupe		Cultivar *		Landrace *	
	*r* ^2^	SD	*r* ^2^	SD	*r* ^2^	SD	*r* ^2^	SD	*r* ^2^	SD	*r* ^2^	SD
0–1	0.73	0.37	0.87	0.26	0.73	0.37	0.84	0.31	0.89	0.26	0.87	0.28
1–2	0.71	0.35	0.92	0.17	0.67	0.39	0.83	0.34	0.92	0.21	0.94	0.15
2–4	0.64	0.40	0.96	0.06	0.65	0.39	0.78	0.35	0.88	0.22	0.86	0.21
4–7	0.59	0.39	0.77	0.32	0.58	0.38	0.78	0.35	0.79	0.31	0.75	0.35
7–10	0.63	0.36	0.80	0.34	0.55	0.37	0.79	0.34	0.83	0.26	0.79	0.35
10–20	0.55	0.36	0.74	0.38	0.44	0.37	0.70	0.38	0.83	0.35	0.68	0.41
20–40	0.49	0.35	0.64	0.43	0.35	0.34	0.63	0.40	0.70	0.40	0.55	0.44
40–70	0.44	0.33	0.70	0.39	0.27	0.32	0.57	0.41	0.69	0.40	0.69	0.40
70–100	0.40	0.32	0.60	0.42	0.22	0.29	0.56	0.39	0.65	0.41	0.58	0.41
100–150	0.39	0.31	0.57	0.43	0.18	0.25	0.50	0.40	0.64	0.41	0.57	0.44
150–200	0.37	0.30	0.52	0.44	0.16	0.23	0.46	0.39	0.58	0.42	0.47	0.45
200–250	0.36	0.29	0.51	0.42	0.14	0.22	0.44	0.38	0.52	0.44	0.51	0.43
250–300	0.35	0.29	0.52	0.44	0.12	0.20	0.45	0.39	0.58	0.41	0.51	0.46
300–1000	0.34	0.28	0.42	0.42	0.10	0.16	0.39	0.37	0.47	0.42	0.40	0.43
1000–1500	0.32	0.27	0.25	0.37	0.10	0.15	0.31	0.32	0.36	0.40	0.23	0.35

SD, standard deviation; * Makuwa.

## Data Availability

Relevant data are available at NCBI’s Sequence Read Archive under accession number PRJNA690179.

## Supplementary Materials

The following are available online at https://www.mdpi.com/article/10.3390/ijms22136722/s1, Figure S1: The homozygote and heterozygote single nucleotide polymorphism (SNP) ratios across the chromosomes, Figure S2: ADMIXTURE results assuming two ancestral populations. Colors represent ancestry components. Stacked bars represent samples. Samples are arranged according to taxonomy as indicated on the *x*-axis, Figure S3: Single nucleotide polymorphism (SNP) markers to discriminate the muskmelon, makuwa, and cantaloupe varieties, Figure S4: Manhattan plots of the genome-wide association study for the phenotypic traits of 1000-seed weight (TSW), length, and width in the melon populations, Figure S5: The candidate genes (A) protein ABIL1 and (B) titin homolog isoform x2 underlying the 1000-seed weight (TSW) variation in melon accessions. Each panel shows the association statistics for TSW in melon accessions, the location of the genome-wide association study (GWAS)-associated region on chromosome 6, and candidate genes and the LD haplotype heatmap for the GWAS region harboring genes and the segregating single nucleotide polymorphisms (SNPs) (S6_875904 and S6_5912593). The presence of genes and the segregating SNPs are indicated by the red cursor in each location of the figure, Table S1: Sampling information and RDA accession numbers of melon varieties, Table S2: Demultiplexing, adaptor trimming, and read mapping, Table S3: Information about homozygote and heterozygote single nucleotide polymorphisms (SNPs) based on genotyping-by-sequencing (GBS) in melon accessions, Table S4: Statistics of genetic variation for the makuwa cultivars and landraces, Table S5: Results of analysis of molecular variance (AMOVA) and F-statistics within the makuwa cultivars and landraces, Table S6: Pairwise distance between single nucleotide polymorphisms (SNPs).
