# Supplementary material for: Genome-Wide SNP Markers for Genotypic and Phenotypic Differentiation of Melon (Cucumis melo L.) Varieties Using Genotyping-by-Sequencing
Source: Int J Mol Sci. 2021 Jun 23;22(13):6722. doi: 10.3390/ijms22136722 (PMC8268568; doi:10.3390/ijms22136722)
Supplement: Supplementary file 1 [file ijms-22-06722-s001.zip › GBS_melon_Manuscript_suppl.Figs_S1-S5.pptx]

## Slide 1
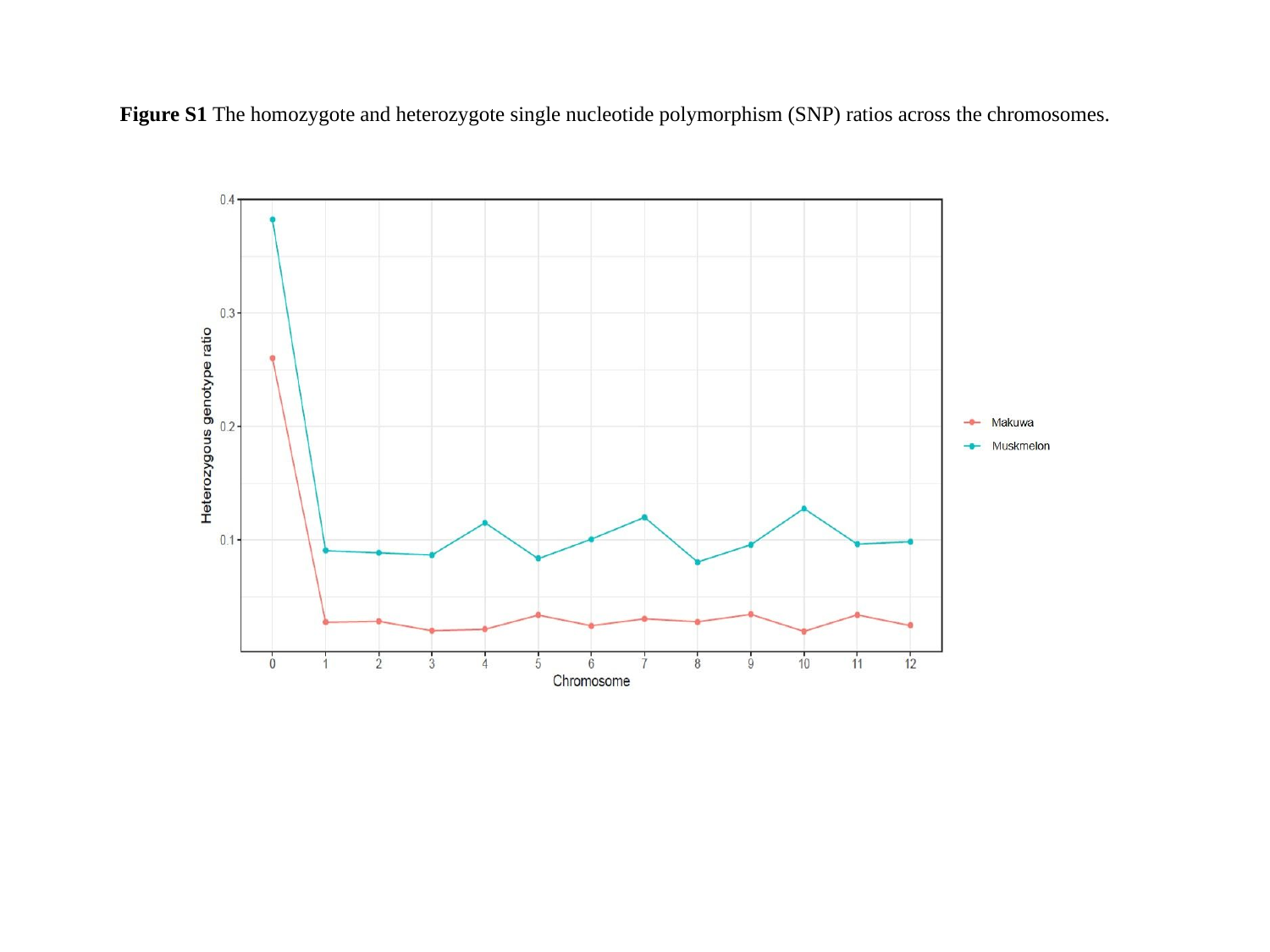

Figure S1 The homozygote and heterozygote single nucleotide polymorphism (SNP) ratios across the chromosomes.

## Slide 2
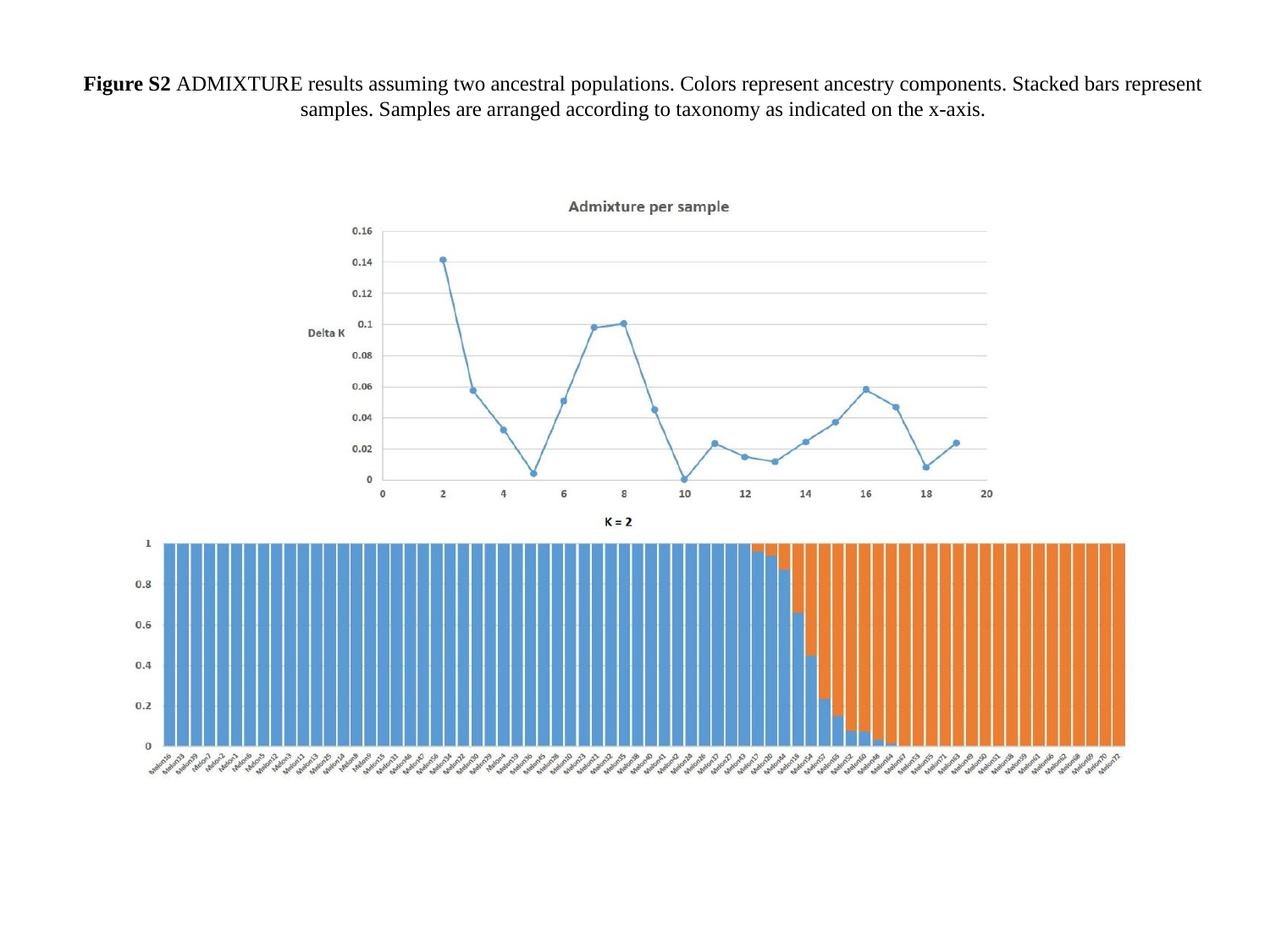

Figure S2 ADMIXTURE results assuming two ancestral populations. Colors represent ancestry components. Stacked bars represent samples. Samples are arranged according to taxonomy as indicated on the x-axis.

## Slide 3
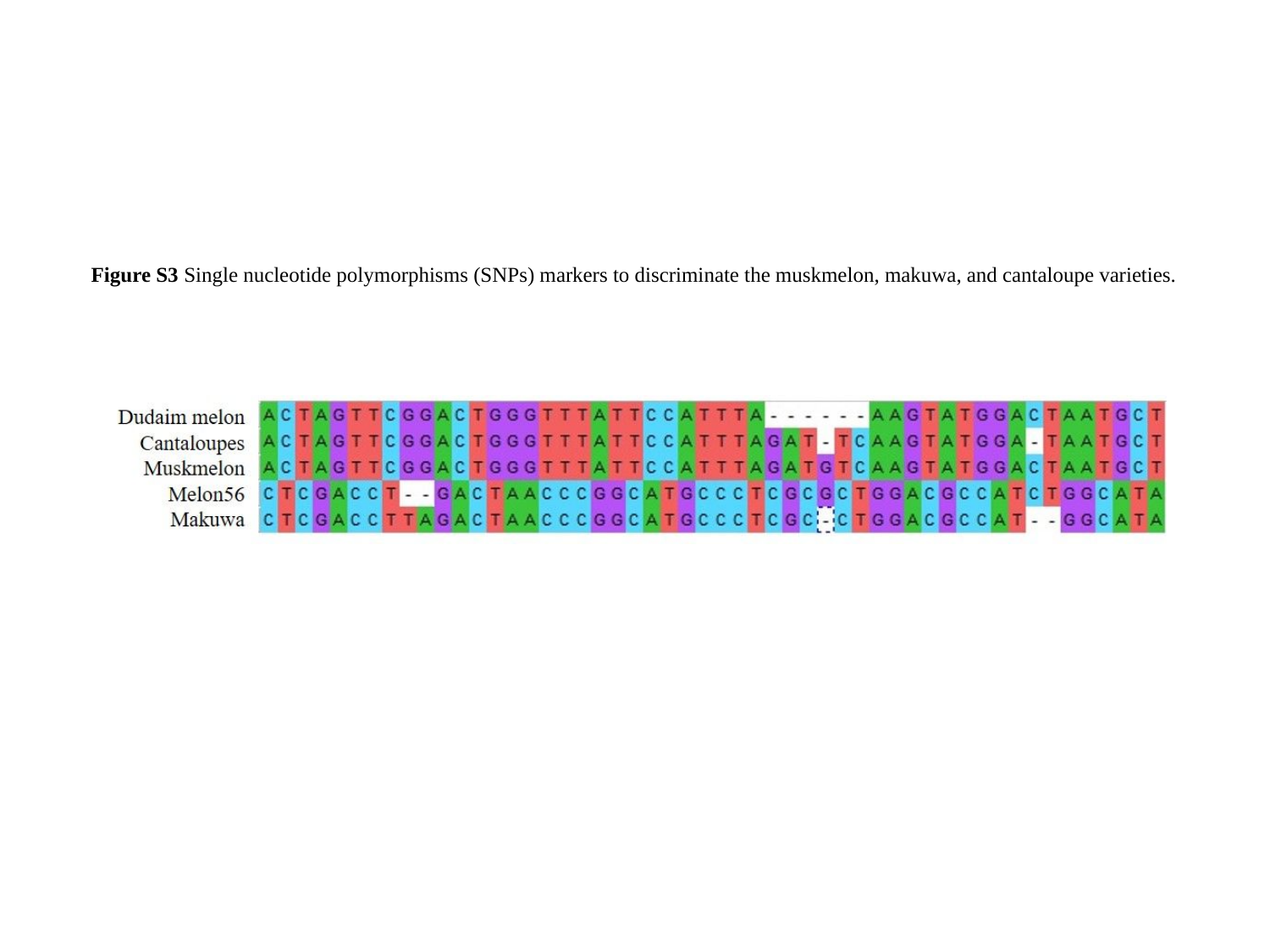

Figure S3 Single nucleotide polymorphisms (SNPs) markers to discriminate the muskmelon, makuwa, and cantaloupe varieties.

## Slide 4
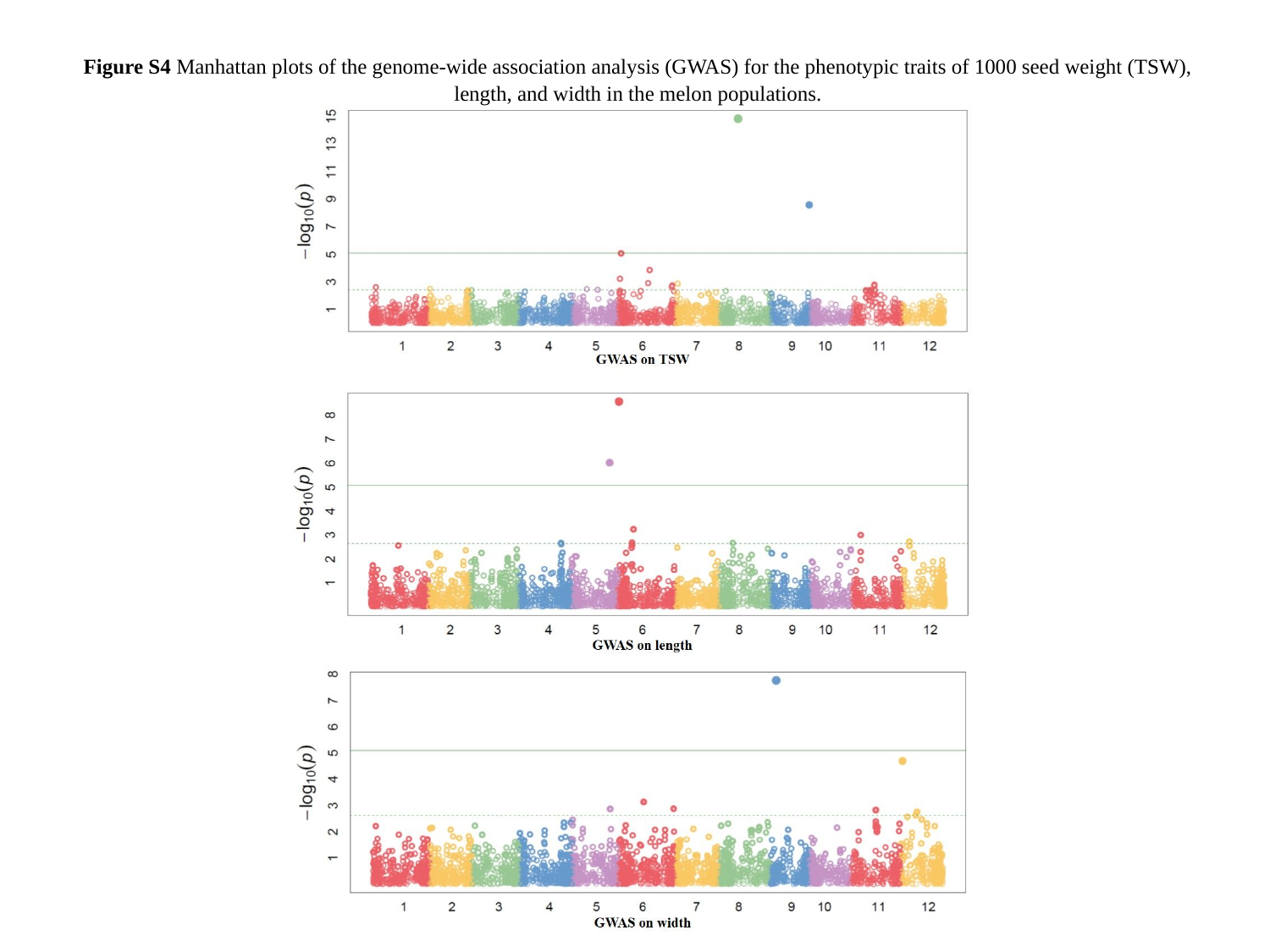

Figure S4 Manhattan plots of the genome-wide association analysis (GWAS) for the phenotypic traits of 1000 seed weight (TSW), length, and width in the melon populations.

## Slide 5
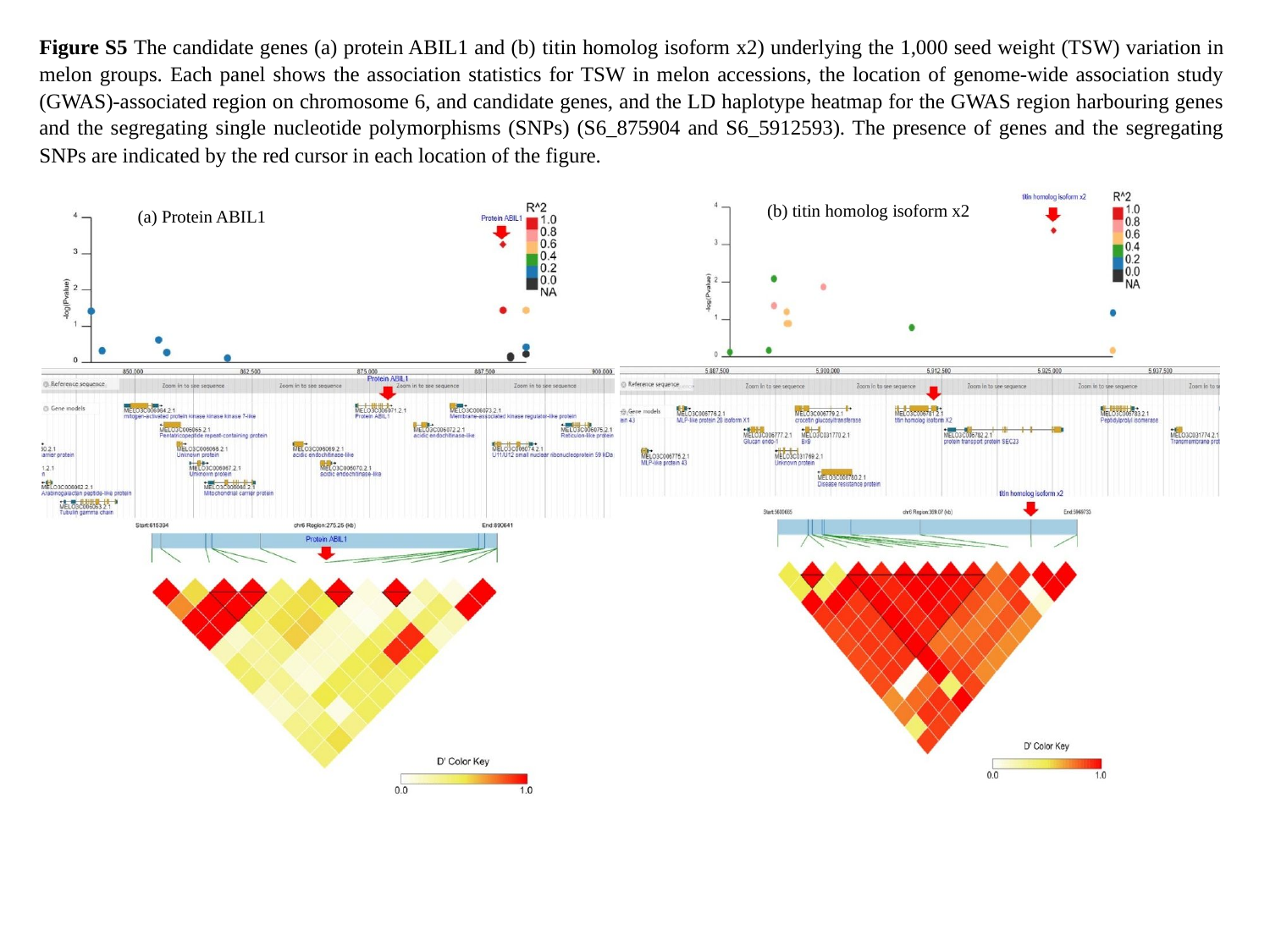

Figure S5 The candidate genes (a) protein ABIL1 and (b) titin homolog isoform x2) underlying the 1,000 seed weight (TSW) variation in melon groups. Each panel shows the association statistics for TSW in melon accessions, the location of genome-wide association study (GWAS)-associated region on chromosome 6, and candidate genes, and the LD haplotype heatmap for the GWAS region harbouring genes and the segregating single nucleotide polymorphisms (SNPs) (S6_875904 and S6_5912593). The presence of genes and the segregating SNPs are indicated by the red cursor in each location of the figure.
(b) titin homolog isoform x2
(a) Protein ABIL1
