# Supplementary material for: Genome-Wide SNP Markers for Genotypic and Phenotypic Differentiation of Melon (Cucumis melo L.) Varieties Using Genotyping-by-Sequencing
Source: Int J Mol Sci. 2021 Jun 23;22(13):6722. doi: 10.3390/ijms22136722 (PMC8268568; doi:10.3390/ijms22136722)
Supplement: Supplementary file 1 [file ijms-22-06722-s001.zip › GBS_melon_Manuscript_Suppl.Tables_S4-S6.pdf]

Table S4. Statistics of genetic variation for the makuwa cultivars and landraces

Variety	N	Na	Ne	I	He	uHe	%P
Cultivars	21.000	0.897	1.111	0.113	0.070	0.072	33.55%
Landraces	26.000	0.700	1.105	0.100	0.064	0.066	23.62%

N, number of individuals; Na, number of alleles; Ne, number of effective alleles; I, Information index; He, expected heterozygosity; uHe, unbiased expected heterozygosity; %P, percentage of polymorphic loci

Table S5. Results of analysis of molecular variance (AMOVA) and F-statistics within the makuwa cultivars and landraces

SV	df	SS	MS	Est. Var.	%	PhiPT
Among Pops	1	493.162	493.162	13.791	7%	0.074
Within Pops	45	7773.647	172.748	172.748	93%	
Total	46	8266.809		186.538	100%	

SV, Source of variation; df, degrees of freedom; SS, sum of squares; MS, mean square; Est. Var., Estimated variance; %, Percentage of variation.

Table S6. Pairwise distance between single nucleotide polymorphisms (SNPs).

	C. melo* L. var. *makuwa		***C. melo* subsp. *melo***	
**Varieties**	**Non-matching nucleotides**	**Pairwise distance**	**Non-matching nucleotides**	**Pairwise distance**
C. melo L. var. *makuwa*	0	0.00%	52	100.00%
C. melo subsp. *melo*	52	100.00%	0	0.00%
C. melo var. *cantalupensis*	50	96.15%	2	3.84%
C. melo subsp. *melo* 56	5	9.61%	49	94.23%
C. melo subsp. *melo* 54	51	98.07%	6	11.53%
